# Creating a resident-centric rehabilitation research team

**DOI:** 10.1186/s12909-022-03167-3

**Published:** 2022-03-12

**Authors:** Annie M. Abraham, Audrie A. Chavez, Aardhra M. Venkatachalam, Samarpita Sengupta, DaiWai M. Olson, Kathleen R. Bell, Nneka L. Ifejika

**Affiliations:** 1 Department of Physical Medicine and Rehabilitation, UT Southwestern Medical Center, 5323 Harry Hines Blvd, 75390 Dallas, TX USA; 2 Ross University School of Medicine, Miramar, FL USA; 3 Department of Physician Assistant Studies, School of Health Professions, UT Southwestern Medical Center, Dallas, TX USA; 4 Department of Neurology, UT Southwestern Medical Center, Dallas, TX USA; 5 Department of Population and Data Sciences, UT Southwestern Medical Center, Dallas, TX USA

**Keywords:** Medical education, Program development, Physiatry, Neurosciences, Research, Residency, Stroke

## Abstract

**Background:**

The 36-month Physical Medicine and Rehabilitation (PM&R) or Physiatry residency provides a number of multidisciplinary clinical experiences. These experiences often translate to novel research questions, which may not be pursued by residents due to several factors, including limited research exposure and uncertainty of how to begin a project.

Limited resident participation in clinical research negatively affects the growth of Physiatry as a field and medicine as a whole. The two largest Physiatry organizations – the Association of Academic Physiatrists and the American Academy of Physical Medicine and Rehabilitation – participate in the Disability and Rehabilitation Research Coalition (DRRC), seeking to improve the state of rehabilitation and disability research through funding opportunities by way of the National Institutes of Health (NIH), the National Institute on Disability, Independent Living and Rehabilitation Research (NIDILRR) and the Patient-Centered Outcomes Research Institute (PCORI). A paucity of new Physiatry researchers neutralizes these efforts.

**Results:**

This paper details the creation of a novel, multidisciplinary Rehabilitation Resident Research program that promotes resident research culture and production. Mirroring our collaborative clinical care paradigm, this program integrates faculty mentorship, institutional research collaborates (Neuroscience Nursing Research Center, Neuroscience Research Development Office) and departmental resources (Shark Tank competition) to provide resident-centric research support.

**Conclusions:**

The resident-centric rehabilitation research team has formed a successful research program that was piloted from the resident perspective, facilitating academic productivity while respecting the clinical responsibilities of the 36-month PM&R residency. Resident research trainees are uniquely positioned to become future leaders of multidisciplinary and multispecialty collaborative teams, with a focus on patient function and health outcomes.

## Introduction

### A need for research structure

There are approximately eighty-three Accreditation Council for Graduate Medical Education (ACGME) accredited residency programs in Physical Medicine and Rehabilitation (PM&R) in the United States. Among the 1,409 active PM&R trainees detailed in the Association of American Medical Colleges (AAMC) 2020 Report on Residents [1, 2], there are opportunities to expand research programs focused on improving the function, mobility, and quality of life for patients with disabling conditions. There are national programs, such as the Rehabilitation Medicine Scientist Training Program (RMSTP), that offer a structured pathway for selected participants to pursue research; though there are limitations regarding the ability to support a large number of resident trainees.

Residents outside of these programs benefit from supportive research environments *within their home institutions* that provide training to spur research innovation. In 2016, Kosik et al. reviewed 1,294 publications demonstrating that 83% of the graduates of physician scientist training programs entered a career in academics; however, the review also emphasized that despite the effort put forth by the National Institutes of Health (NIH) and the scientific community, these programs fell short in both the number and the quality of physician scientists produced [3].

To combat the shortfall, several institutions have taken steps to create resident research programs. One such example is a PM&R resident research track at the University of Pittsburgh which operates in concert with the RMSTP. Applicants to the track undergo a competitive selection process as early as their first year of residency (PGY-1) including interviews with the Research Track Director, essays, and letters of support. Once in the program, residents complete online research modules, one-month research rotations, and bi-annual progress meetings, and continued involvement in the program is contingent upon acceptance into the RMSTP [4].  In addition, the University of Pittsburgh Internal Medicine residency program implemented the Leadership and Discovery Program (LEAD) to guide and support non-research track residents by setting a programmatic expectation to participate in a scholarly project. Three binary metrics were used to measure scholarly productivity: presentations at a regional, national, or international scientific meeting, publications in a peer reviewed journal, and completion of one or more scientific presentations. Notably, resources to support individual projects, including statistical support, were provided by individual mentors and not by LEAD or the Department of Medicine. Furthermore, the publication did not describe any other departmental or interdisciplinary collaboration or support for mentors or participants [5].

### Barriers to resident research

Although the ACGME mandates scholarly activity for residents, metrics for success are not well-defined. The current ACGME PM&R residency scholarly activity requirement specifies that each resident should “demonstrate scholarship through at least one scientific presentation, abstract, or publication” [6], and it is possible that this requirement may further develop to specify a peer-reviewed publication or a regional or national conference presentation. Numerous factors have been used to determine the success of a resident research program including protected time for research activities, supplementary research curricula, and specialized research tracks. However, a systematic review by Stevenson et al. (2017) showed that programs need to provide increased structure and that resident scholarly activity success is directly linked to the concordance of program pathways with resident goals [7].

Despite a PM&R scholarly activity requirement, barriers that impede resident participation and success in research include lack of knowledge and skill to design, execute, and disseminate results of a research project as well as lack of protected time, interest, mentorship and faculty support, opportunities for collaboration, and funding [8]. By the virtue of their training being primarily focused on developing clinical skills, residents often have limited experience and knowledge in research design and implementation. In addition, the tenets of team science and collaborations, institutional and federal policies, and guidelines for the ethical conduct of research, budgeting, and grant seeking are not taught at any level. In this paper, we describe the experience of a multi-disciplinary resident-centric rehabilitation research team designed to overcome the aforementioned barriers. The research team is innovative and resident-driven, with ample support from faculty, collaborators, the department, and the institution.

### Creating a research team

**Fig. 1:**
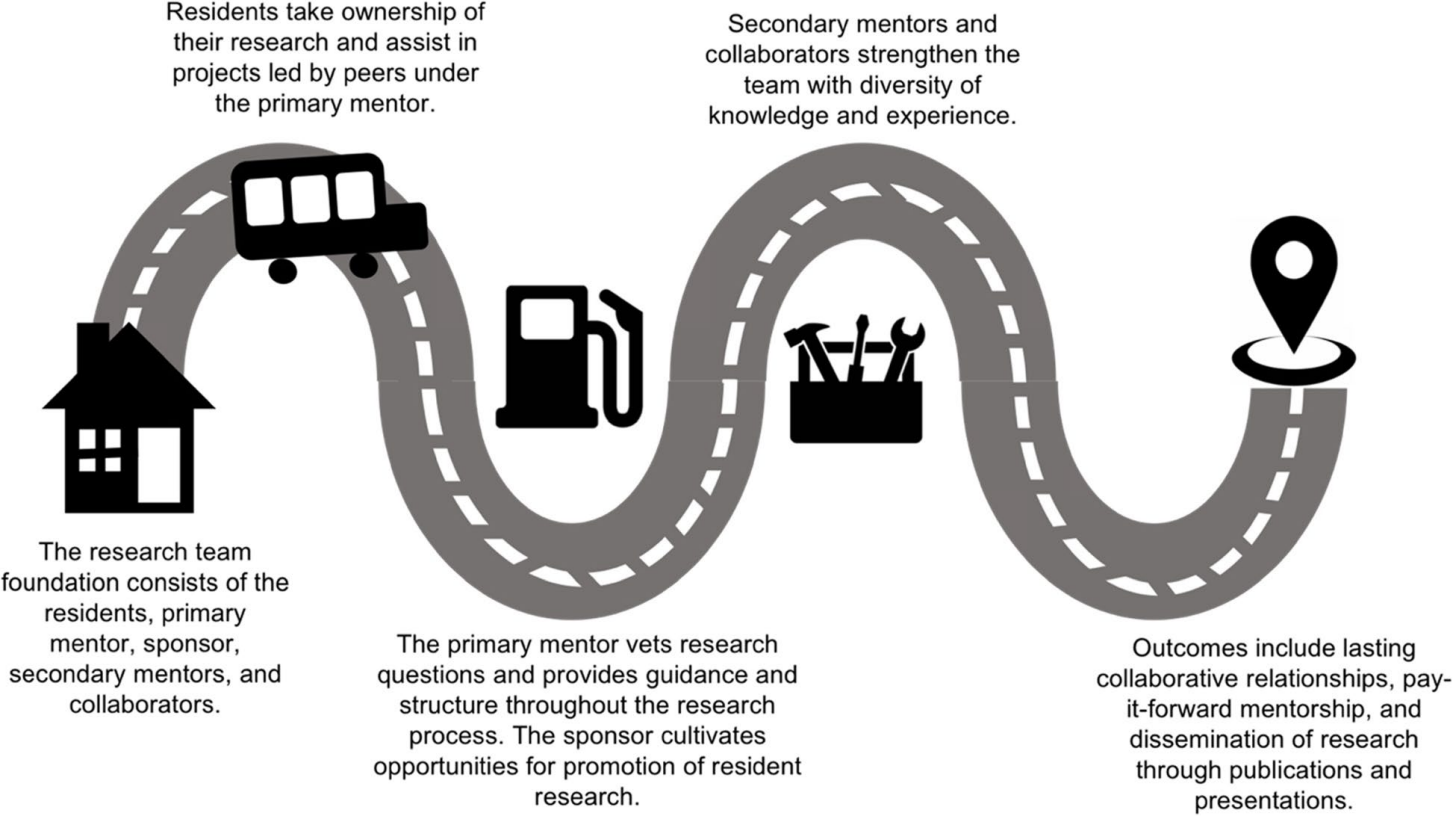
Resident research program structure

The research team is the foundation of the resident research program experience (Fig. 1). The research team focused in the Department of PM&R at the University of Texas (UT) Southwestern Medical Center consists of residents, each of whom is assigned a primary mentor, a sponsor, secondary mentors, and collaborators.

The residents develop their own research question and design the project with guidance from the primary mentor. Residents in the team synergistically lead their own projects and collaborate on their co-residents’ projects. This dynamic is important as team members work cohesively to implement the project and collect data despite variations in individual workload.

The primary mentor is a faculty member with ample research and mentoring experience who serves as the first contact for residents. The primary mentor vets the research questions put forth by the residents and helps shape their projects by providing suggestions and guidance. She also facilitates the more technical aspects of launching a project, including educating residents on processes involved, such as required trainings and compliance approvals. All the while, she leads regular meetings every other week in which every member of the team shares and provides updates on their projects. In the first iteration of this program, Dr. Nneka Ifejika served as primary mentor.

The sponsor is an established academician with an extensive track record in publishing research and in research funding who holds a high leadership position within the department. The sponsor builds up a research presence in the department, including cultivating opportunities for resident research to be promoted within the department as well as obtaining funding for resident research both within the institution and through external sources. She is in frequent contact with the primary mentor and provides a fund of knowledge on opportunities that exist both within and outside of the department. Dr. Kathleen Bell served in this capacity.

Secondary mentors and collaborators from both within and outside of the department strengthen the team in terms of diversity of knowledge and experience. Incorporating these members into a collaborative network enhances research progress through fresh perspectives as well as connections to resources. Dr. DaiWai Olson served as secondary mentor and Dr. Samarpita Sengupta as a collaborator.

### Pillars of resident involvement

#### Resident initiative

In order to promote resident research involvement, it is important for residents to define their scholarly interests, including specific research interests within PM&R as well as their general career goals. With these in mind, they can identify mentors who share these academic interests and can guide them both within the realm of research and in the shaping and development of their career. In this team’s experience, the residents raised questions related to evidence-based medicine and systems-based practices as it pertained to their clinical experiences. This ultimately led the residents to identify interests in health disparities and stroke rehabilitation research and were thereby able to connect with a mentor with similar interests.

#### Visibility and promotion

The PM&R residency consists of three years of specialty training (PGY-2 through PGY-4) after a preliminary internship year which may limit ability to see projects through to completion and timely exposure to mentors whose professional and research goals align with those of the resident. Therefore, it is helpful for residents to connect with a mentor as early as possible to have adequate time to carry out a meaningful project. In order to achieve an early mentorship relationship, it is beneficial for research-focused faculty to have opportunities to meet and engage with residents. In this team’s experience, the primary mentor led weekly stroke rounds during two clinical rotations in the PGY-2 year. She also played an active role in teaching a stroke rehabilitation resident didactic series as well as mentoring resident-led journal clubs. Through intentional clinical, educational, and mentoring experiences, these residents were able to connect with the primary mentor early in their first year of PM&R training.

#### Consistent mentorship

The strongest pillar of resident involvement is longitudinal mentorship. The research team has a standing bi-weekly meeting in which research project updates are shared, and together the group collaborates on next steps prior to meeting again. All members of the team are committed to attending these meetings and hold an invested interest in ongoing projects within the team with a united ambition to advance team members’ projects and careers. As a result, continued progress is ensured, and members are kept accountable. Additionally, these mentoring meetings allow opportunities for the primary mentor to offer meaningful life and career guidance, foster independence, and maintain effective and inclusive communication across differences. With residents at various levels of training, these conversations also encourage ongoing peer mentorship between senior and junior residents. Comprehensive mentorship is the foundation of a productive and cohesive research team.

#### Launching opportunities

In order to cultivate an environment that promotes research, support, and opportunities for funding, collaboration and celebration are necessary. At UT Southwestern Medical Center, the PM&R department hosts an annual event titled “Shark Tank” in which residents and postdoctoral fellows present their research ideas with the objective of obtaining departmental funding. Research ideas presented can be at any stage of development, and newcomers are welcomed to participate and share their ideas. This event also offers an opportunity for constructive feedback from faculty and peers for the resident-led research projects.

**Fig. 2:**
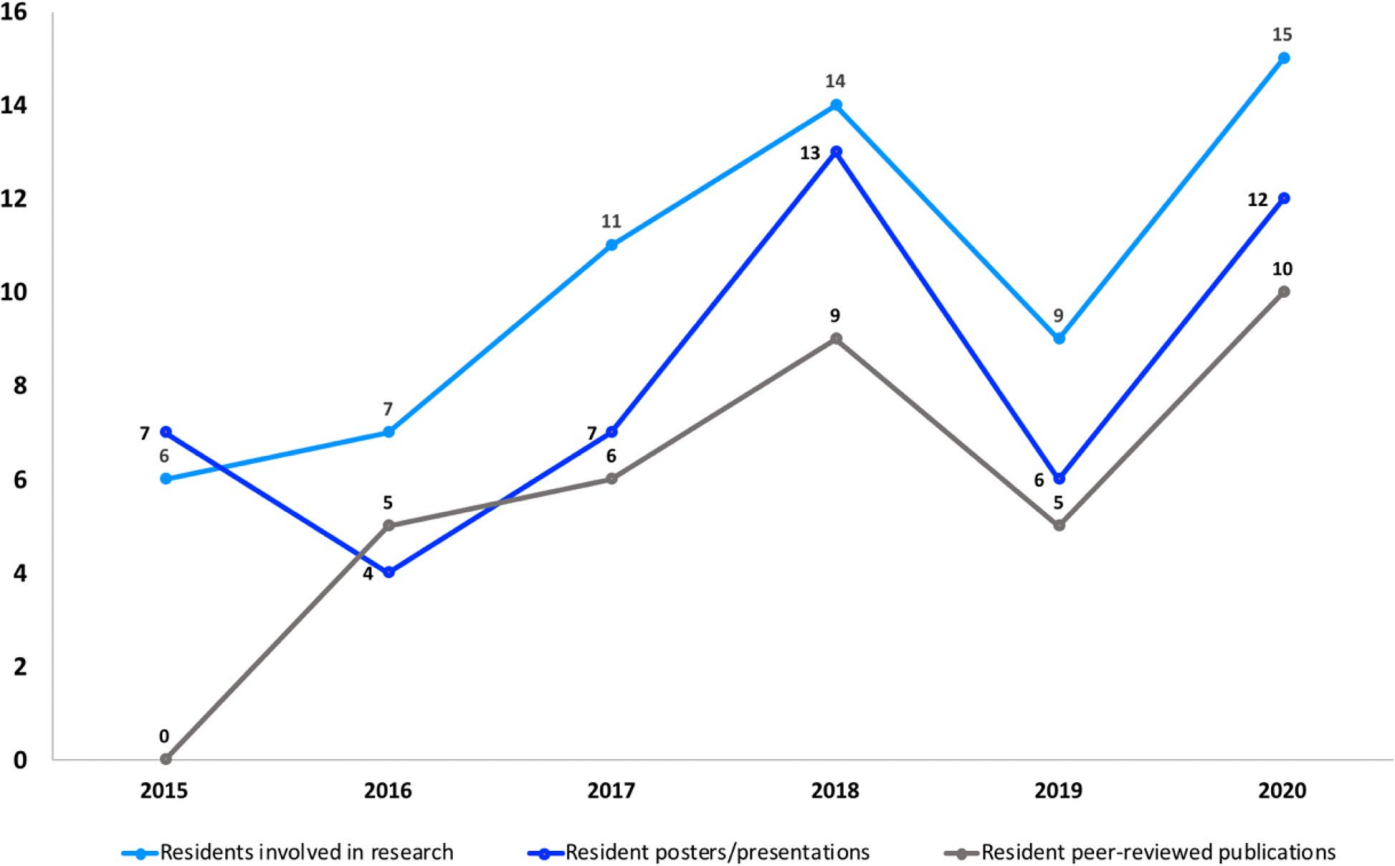
Resident research involvement and productivity from 2015 to 2020

Notably, since the initiation of the “Shark Tank” competition in 2016, the number of peer-reviewed publications by a total of 36 residents has increased from zero in 2015 to a total of 35 between 2016 and 2020 (Fig. 2). As shown in Fig. 2, there was a positive trend in the number of resident posters and presentations at conferences as well as overall involvement in research during this period as well. More specifically, in the five years after the initiation of “Shark Tank”, approximately 1 in 2 graduating residents published peer-reviewed papers, demonstrating a considerable increase from the 1 in 4 residents in the five years prior to the first competition.

Research publications and presentations are highlighted in a quarterly PM&R CONNECTion newsletter, which increases academic visibility and fosters interdepartmental collaborations [9]. In addition, there are several other departmental events in which resident research is highlighted and emphasized through project presentations, including an annual interdepartmental conference hosted by the PM&R department known as Scientific Day as well as through annual senior resident capstone project presentations. There are awards to spotlight top resident research projects through these aforementioned avenues as well. Along with this, the primary mentor, secondary mentor, and collaborators alert residents to presentation opportunities outside of the PM&R department. These opportunities allow residents to gain valuable experience in disseminating their research findings.

#### Continuing collaboration

In this team’s experience, there were ample opportunities to learn from and to become involved in both interdepartmental and interinstitutional resident research. This was made possible by the extensive collaborative network cultivated by the primary mentor, who connected residents with staff and resources that allow them to advance in research-related and professional goals, often through the assistance of secondary mentors and collaborators. As a result, residents gathered experience in collaborative relationships as well as further exposure to research structure and multi-disciplinary or multi-center project designs.

**Fig. 3:**
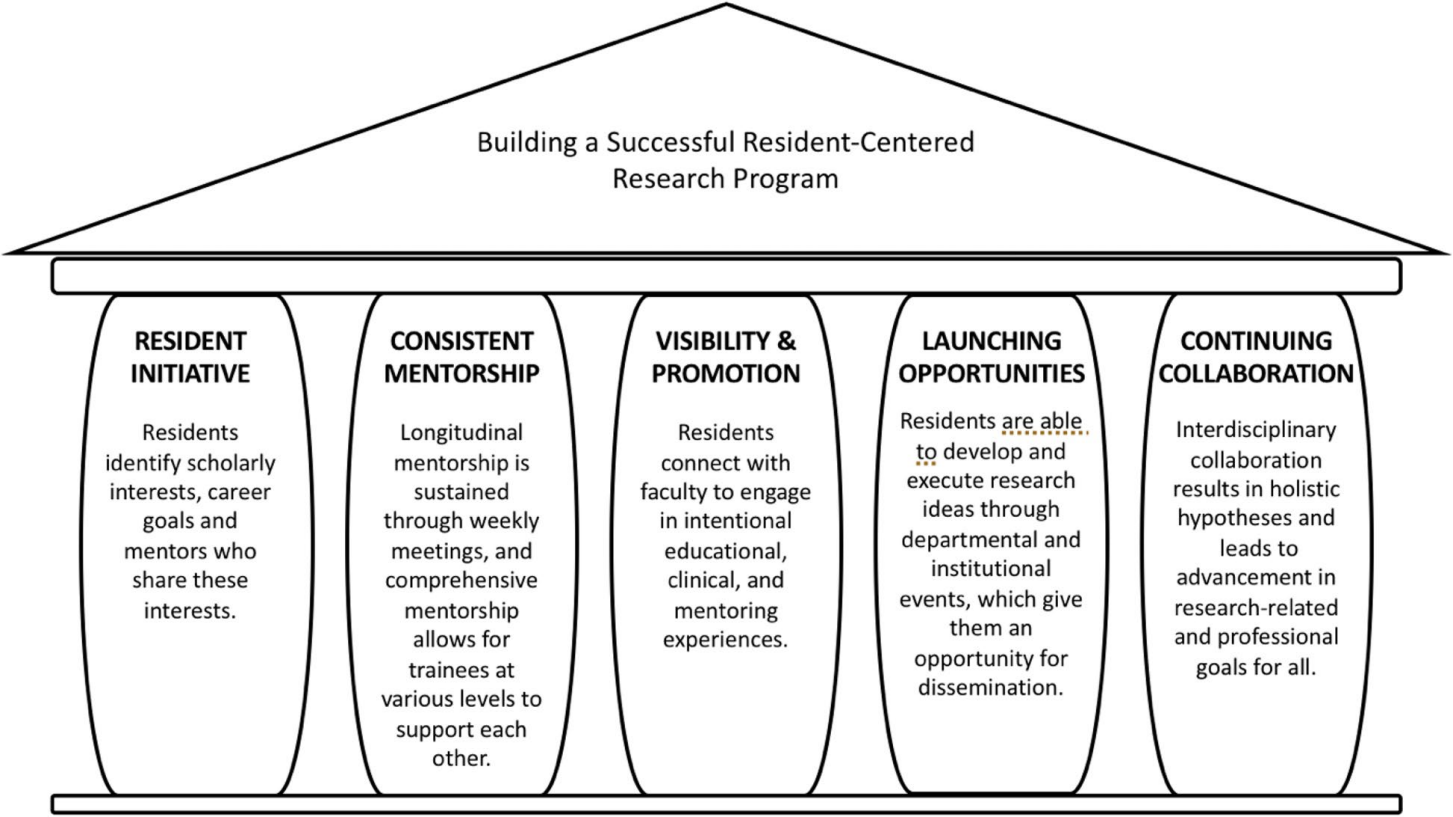
Pillars of resident involvement in building a successful resident-centered research program

Through effective mentoring, strong departmental and institutional support, and avenues for interdisciplinary collaboration, the UT Southwestern Medical Center PM&R resident-centric research program (Fig. 3) mirrors the academic institutional model for multidisciplinary clinical work and cultivates professional practices that bridge bench to the bedside.

### **Funding**

#### **Opportunities**

For the UT Southwestern Resident Rehabilitation Research program, research funding is provided through two mechanisms. Funds from an endowed professorship to the Department Chair are used to award selected candidates from the “Shark Tank” competition and support research infrastructure, including dedicated staff. Next, the Giving Back to Promote Residency Development (GO-PMR) fund, which was established by and maintained through generous donations, is managed by the Office of Development and Alumni Relations and is used for specialized resident education funding, including costs associated with presentations at national and international conferences. The kickoff event for GO-PMR was a large-scale fundraising event in 2019; the proceeds were matched by a donation from the President at UT Southwestern Medical Center.

#### Additional programs and resources

In addition to the resources provided by the Department of PM&R, the resident research team also has access to additional resources offered across the institution.

The resident research team has access to Research Development services through the primary mentor. Research Development (RD), not to be confused with Research Administration, is a set of catalytic and proactive services designed to increase research productivity in institutions [6–9]. RD services range from idea development, collaboration and team building, grant seeking, and proposal development to strategic initiatives like curriculum development and seed funding programs as well as larger institution-wide initiatives aimed at boosting research capacity and funding.

The Neuroscience Research Development (NeRD) Office at UT Southwestern Medical Center offered research development services to faculty, students, fellows, and residents engaged in neuroscience research. NeRD was established in 2015, and until 2020, offered one-on-one guidance and mentorship to researchers starting their research careers as well as management of large multi-institutional, multi-researcher projects that were spearheaded by more established researchers. NeRD also engaged in creating and implementing strategic initiatives to bridge gaps in training, including the Resident Research Curriculum. In 2021, commensurate with restructuring, NeRD was dissolved and a few components were moved to other parts of the university.

#### Resident research didactics

The Resident Research Curriculum started by the Department of Neurology consists of 4 to 5 lectures on topics such as Introduction to Research Design, Practical Aspects of a Human Research Study, Basic Statistics, Research Communication, and Publications. The information from these presentations is applied by the residents through the design of a meaningful research project, with a typical timeline of 24 months for feasibility. Residents are paired with an experienced mentor who guide the projects on a day-to-day basis. Throughout their projects, the residents present works-in-progress seminars, at least once a year, where they are able to troubleshoot issues with input from other faculty members and their peers. These projects culminate in a Research Day hosted by the Department of Neurology where final year residents showcase their research as posters or podium presentations. Many of these projects are eventually disseminated through peer-reviewed publications. These presentations are also open to PM&R residents to attend.

#### Research resources

In addition to providing a platform for research education, NeRD offered residents introduction to institutional, federal, and state resources to enable success in their research projects. NeRD connected residents to the UT Southwestern Library Services that offers resources to assist with literature review, reference management, institutional programs and software, and funding databases. NeRD also connected residents with biostatisticians who could help them design and analyze projects as well as clinical coordinators who could help with Institutional Review Board (IRB) applications and provide other administrative assistance.

Research Development services are often times provided at an institutional level, but they are also found at college and department levels. It is in the resident’s best interest to establish contact with research development professionals at their institutions and take advantage of the many opportunities that are made available, which is best achieved through a network of mentors and collaborators.

### Concepts for success

With the success of the Resident Research Program, we have identified the concepts highlighted below that would guide institutions aiming to develop such programs.

#### Collaboration and research teams

Integration into the research culture follows a similar pattern to integration into any culture. Some members will integrate quickly and easily and others may never fully integrate as part of the research team. A primary aim of team building is to identify the relative strengths and weaknesses of each team member. Residents arrive to a PM&R site with diverse backgrounds from different schools of medicine and college experience, each with different goals and emphasis on research.

The silo concept has long been known to be a barrier to integration. The phrase ‘working in silo’ originates from old grain silos which were large pits lined with stone. When someone was working in the silo they could not hear cries for help; and if injured, their cries could not be heard. Yet, silos develop in every aspect of a culture, and team leaders must aim to identify them and determine ways to integrate silos. A regularly scheduled research meeting facilitates a time during which all team members can learn about the research activities taking place throughout the department. This also facilitates integration by providing a time during which residents can volunteer to work on research projects.

#### Feasibility of research projects

Providing mentoring during the design phase of the study is fundamentally important. Not all study designs are practical for a resident to complete during residency (e.g., a clinical trial with 5-year followup). Some residents who have research experience may wish to continue their own line of research, though many residents will benefit from participating in ongoing trials. Making an early assessment of the individual goals and abilities helps align residents with faculty mentors. For example, the resident who wants or needs writing experience may benefit from joining a team that has just completed the data collection phase, while a resident who needs experience in study design would benefit from joining a team that has not yet submitted to the IRB.

#### Research culture

In addition to providing resources highlighted above, programs and departments can work to create cultures that promote research and innovation. For instance, asking residents to start working on research only after all their ‘real work’, or clinical work, is done sends the message that research is not central to the mission. Knowing that faculty must incorporate research into the daily routine and knowing that one mission of education is to teach survival skills for the real world, it is incumbent on the team to emulate and teach effective time management techniques to residents so that they can participate in research during normal working hours. One method is to add research time to the daily schedule.

Future steps include implementation of this concept with other teams in different areas of rehabilitation and incorporating non-research clinical attending physicians as collaborators to enhance their comfort in supporting trainees in research endeavors. In fact, these ideals are in the initial phases at this institution within other areas of Physiatry outside of stroke rehabilitation. Research “families” with identified primary mentors and sponsors have been established involving both faculty and residents of varying levels of research experience. As part of this, faculty development seminars have been initiated to address the need for instruction on formulating research questions. The adaptation of the resident-centric rehabilitation research team model to other areas within PM&R is expected to contribute to continued growth and emphasis on the research culture of the department.

As a result of implementation of these strategies, outcomes would include the development of lasting collaborative relationships, mentorship that trains the next generation of research leaders, and scientific products in terms of oral presentations and peer-reviewed publications, thereby overall moving the needle of research in Physiatry forward.

## Conclusions

Physiatry as a specialty is a brain trust of clinicians, focused on improving the function of patients with disability. In order to advance the field of PM&R and medicine overall, there is a need for residency programs to pay special attention to the role of resident research within the 36-month training period. Establishing responsibilities of primary and secondary mentors, sponsors, and collaborators, as well as structuring communication, incorporating novel research ideas, and providing opportunities to join ongoing projects are all important parts of successful research collaborations. Departments are encouraged to establish funding specifically purposed for readily accessible resident research support, and deliverables, including publications, should be required. The resident-centric rehabilitation research team has formed a successful research program that was piloted from the resident perspective, facilitating academic productivity while respecting the clinical responsibilities of the 36-month PM&R residency. Resident research trainees are uniquely positioned to become future leaders of multidisciplinary and multispecialty collaborative teams, with a focus on patient function and health outcomes.

## Data Availability

The datasets used and/or analyzed during the current study are available in the PM&R CONNECTion departmental newsletter repository, https://www.utsouthwestern.edu/education/medical-school/departments/physical-medicine/newsletters.html.

## Abbreviations

ACGME: Accreditation Council for Graduate Medical Education
GO-PMR: Giving Back to Promote Residency Development
IRB: Institutional Review Board
NeRD: Neuroscience Research Development
NIH: National Institutes of Health
PM&R: Physical Medicine and Rehabilitation
RD: Research Development
RMSTP: Rehabilitation Medicine Scientist Training Program
UT: University of Texas
